# Targeting MicroRNAs in Cancer Gene Therapy

**DOI:** 10.3390/genes8010021

**Published:** 2017-01-09

**Authors:** Weidan Ji, Bin Sun, Changqing Su

**Affiliations:** Department of Molecular Oncology, Eastern Hepatobiliary Surgery Hospital & National Center of Liver Cancer, Second Military Medical University, Shanghai 200433, China; jiweidan326@163.com (W.J.); sunbin05301984@aliyun.com (B.S.)

**Keywords:** cancer, microRNA, gene therapy, oncogene, tumor suppressor gene

## Abstract

MicroRNAs (miRNAs) are a kind of conserved small non-coding RNAs that participate in regulating gene expression by targeting multiple molecules. Early studies have shown that the expression of miRNAs changes significantly in different tumor tissues and cancer cell lines. It is well acknowledged that such variation is involved in almost all biological processes, including cell proliferation, mobility, survival and differentiation. Increasing experimental data indicate that miRNA dysregulation is a biomarker of several pathological conditions including cancer, and that miRNA can exert a causal role, as oncogenes or tumor suppressor genes, in different steps of the tumorigenic process. Anticancer therapies based on miRNAs are currently being developed with a goal to improve outcomes of cancer treatment. In our present study, we review the function of miRNAs in tumorigenesis and development, and discuss the latest clinical applications and strategies of therapy targeting miRNAs in cancer.

## 1. Introduction

MicroRNAs (miRNAs)—a group of small endogenous non-coding functional RNAs—are approximately 18–22 nucleotides in length and widespread in plants and animals [1]. Studies have demonstrated the significance of miRNA biosynthesis and regulatory function in maintaining cellular homeostasis [2]. miRNAs are transcribed by RNA polymerase II, from initial processing to final maturing [3,4,5]. They are then incorporated into the RNA-induced silencing complex (RISC) together with Argonaute to silence target messenger RNA (mRNAs) usually through imperfect complementary base pairing to the 3′-untranslated region [6,7]. A single mRNA may possibly be targeted by multiple different miRNAs with variable efficiencies. Conversely, a single miRNA may target more than one mRNA. Through their binding to target mRNA sequences, miRNAs have various biological functions. They have an ability to inhibit or promote the expression of many related genes, which can affect several cell-signaling pathways essential to tumor development and progression, such as cell proliferation, differentiation, mobility and apoptosis [8,9]. A global reduction or increase of mature miRNAs is observed in cancer and involved in cancer biological behaviors, which has made miRNAs attractive candidates for cancer therapy.

## 2. miRNAs and Cancer

Carcinogenesis is a multistep process. Normal cells experience genetic changes to promote cells through pre-malignant initiation into malignant status. Microarray expression data demonstrated that the aberrant miRNA expression is a common event in cancer [10,11,12]. Importantly, studies featuring miRNA over-expression or ablation on mouse models demonstrated the correlations between miRNAs and cancer development [13,14]. Increasing evidence suggests that miRNAs might play a large and unanticipated role in the occurrence and development of human cancer. A study on a genome-wide basis by mapping 186 miRNAs found that miRNAs were frequently located at fragile sites, minimal regions of heterozygous loss or amplification, or common break-point regions in human cancers [15]. Besides the structural and genetic alterations, the epigenetic silencing of miRNAs genes by DNA promoter hypermethylation or histone hypoacetylation has been clarified in some solid tumors and hematological malignancies [16,17,18].

With the development of genomics, miRNA expression profiles between tumor tissues and normal tissues could be rapidly established through high-throughput technologies such as gene chips, real-time PCR, etc. Dysregulation of miRNA expression has been confirmed in most tumors [19,20]. The up-regulated miRNAs in tumor cells are commonly considered to be oncogenic miRNAs (oncomiRs), which can silence the tumor suppressor genes. miR-21 is a very widely studied oncomiR and has been reported at high expression levels in glioblastoma [21], pancreatic cancer [22], breast cancer [23] and colon cancer [24]. It exerts an antiapoptotic effect by targeting the tumor suppressors such as phosphatase and tensin homolog (PTEN) and programmed cell death 4 (PDCD4) [25,26]. Conversely, some miRNAs, which are often down-regulated in most cancers, can inhibit tumor progression and are termed tumor suppressor miRNAs. These miRNAs target mRNAs of some oncogenes and inhibit the carcinogenic effect by repressing the translation of oncogenic mRNAs [27]. For example, miRNAs are frequently lost in cancer, such as miR-15/miR-16 in chronic lymphocytic leukemia [20]. The miR-15a/miR-16-1 cluster can directly interact with bcl-2 mRNA and inhibit its protein translation, which induces apoptosis of leukemic cells. Loss of miR-15a/miR-16-1 results in an inhibition of leukemia cell apoptosis [28]. Tumorigenesis and development are usually associated with the down-regulation of tumor suppressive miRNAs and the up-regulation of oncomiRs.

Over-expression or down-regulation of some specific miRNAs in different tumors makes them to be potential therapeutic targets. The circulating miRNAs released from their producer cells are novel non-invasive biomarkers in cancers. Several studies detected the circulating miRNAs in cancer patients and discussed their potential relationship to the primary tumors [29]. The circulating miRNAs could be detected in body fluids (blood, urine, tears, saliva, seminal fluid, cerebrospinal fluid, and extracellular fluid) in a fairly stable form and are considered to be valuable in diagnosis and in evaluating prognosis and monitoring treatment response [30,31]. It was reported that the levels of tumor-suppressor miRNAs increased in circulation and are involved in immune responses [32]. Extracellular miRNAs released from normal or tumor cells may function as mediators of paracrine or endocrine signaling pathways among different kinds of tumor cells [33,34,35]. In conclusion, miRNAs may be used for clinical applications in cancer management, not only in tumor diagnosis, but in evaluating malignant potential or therapeutic efficiency, and in monitoring tumor recurrence and progression.

## 3. Strategies of miRNA-Based Cancer Gene Therapy

Intracellular miRNAs bind to the mRNAs of target genes with complementary sequences to induce mRNA degradation or inhibit mRNA translation, thereby exerting their role as post-transcriptional regulators of target genes [36]. Abnormal expression of miRNAs is closely related to cancers. For the purpose of correcting abnormal miRNA expression, miRNA-based gene therapy is becoming a new target strategy for malignant tumors [37]. During recent years, the strategies of miRNA-based tumor treatment are mainly as follows: (1) To inhibit proliferation or induce apoptosis of tumor cells by importing exogenous miRNAs, which are tumor suppressor miRNAs and down-regulated in tumor tissues. For example, the chemically synthesized miRNA mimics are used to imitate endogenous mature double-stranded miRNA with the aim to restore/enhance endogenous miRNA function [38,39]. Construction of viral vectors (adenoviral, lentiviral and retroviral vectors), which express specific miRNAs, could enhance the function of miRNAs in tumor cells; (2) To inhibit the function of miRNAs, which are oncogenic miRNAs and over-expressed in tumors, by applying the antisense oligonucleotides (ASOs) strategy, including anti-miRNA oligonucleotides (AMOs), miRNA antagomirs, locked-nucleic-acids antisense oligonucleotides (LNAs), miRNA sponges, multiple-target anti-miRNA antisense oligodeoxyribonucleotides (MTg-AMOs), miRNA-masking and nanoparticles [40,41,42,43]. LNAs and AMOs are the main types of ASOs that could inhibit miRNA target genes based on complementary base pairing. Antagomir—a small synthetic RNA and complementary to the specific miRNA target—is modified to make it more resistant to degradation [44,45]. Recent studies reported a tumor treatment strategy that simultaneously disturbs the function of multiple oncogenic miRNAs (miRNA sponges). This strategy used a tumor-selective replicating viral vector to mediate the expression of an artificially designed interfering long non-coding RNA (lncRNA), which comprises the binding sites of multiple oncogenic miRNAs and thus neutralizes oncogenic miRNAs in cancer cells, fully protecting the function of tumor suppressor genes and exhibiting an effective anti-tumor effect in vitro and in vivo [46,47]; (3) Artificial miRNA, designed to target single or multiple malignant tumor phenotype-related genes, provides a new therapeutic strategy for cancers [48,49]. The strategy used natural miRNA precursor (pre-miRNA) structures, and specifically interfered with target gene expression by replacing core sequences of pre-miRNA with complementary sequences of target genes. Artificial miRNAs have a stronger silencing effect on target genes compared with short hairpin RNAs (shRNAs); they are regulated by polymerase II promoter to achieve tissue-specific or regulatory expression. The most important feature is its high safety, low toxicity, less effect with cell endogenous RNA interfering (RNAi) and off-target [50,51,52,53].

Until now, some studies of tumor gene therapy targeting miRNA have obtained anti-tumor effects in vivo and in vitro, and laid a foundation for the safe and effective application for tumor patients (Table 1).

## 4. Therapy Targeting miRNAs in Human Cancers

The potential of miRNAs as treatment targets in cancers has been explored by many studies. The therapeutics strategies either introducing tumor suppressor miRNAs or blocking oncogenic miRNAs have developed rapidly in recent years.

### 4.1. Breast Cancer

Breast cancer is a malignant disease threatening the health of women worldwide due to its high capability of recurrence and metastasis. A growing number of studies have demonstrated that miRNAs play critical roles in the development of breast cancer. Romero-Cordoba et al. found that 113 miRNAs showed higher expression and 17 miRNAs were down-regulated in breast tumors compared to the normal adjacent tissue [134]. Furthermore, differential expression of miRNAs has been tightly linked with a high incidence and mortality of breast cancer. It has been well documented that miR-892b expression is obviously down-regulated in human breast cancer specimens. Over-expression of miR-892b by its mimics in breast cancer cells significantly decreased tumor growth, metastatic capacity, and induced angiogenesis in vitro and in vivo, which was mediated by attenuating nuclear transcription factor kappa B (NF-κB) signaling pathway [135]. miR-155 is usually considered to be an oncogenic miRNA in breast cancer. miR-155 antisense oligonucleotide (miR-155 ASO) was synthetized and transfected into MDA-MB-157 cells, the cell proliferation was remarkably inhibited and cell apoptosis was increased [118]. In addition, the use of artificial miRNAs (amiRNA) provides a new strategy for breast cancer therapy. A novel amiRNA, miR p-27-5p, which targets the 3′-untranslated region (3′-UTR) of cyclin-dependent kinase 4 (CDK4) mRNA, was introduced into breast cancer cells. This study revealed that cell proliferation was inhibited and cell cycle was arrested through down-regulation of CDK4 expression and suppression of retinoblastoma protein (RB1) phosphorylation [136]. Liang et al. constructed an amiRNA by inserting a double-stranded miRNA gene against a C-X-C motif chemokine receptor 4 (CXCR4) into a miR-155-based RNAi expression vector, which exhibited a reduced expression level of CXCR4 and a suppressed migration and invasion in breast cancer cells [137].

### 4.2. Hepatocellular Carcinoma

Similar to other malignancies, the pathogenesis of hepatocellular carcinoma (HCC) is a complex with contribution of genetic and epigenetic changes. MiRNAs have been implicated in HCC metastasis. Zhou et al. found that miR-625 was consistently down-regulated in HCC specimens and its re-expression in HCC cells effectively suppressed cell migration and invasiveness by regulating the insulin-like growth factor 2 mRNA-binding protein 1 (IGF2BP1)/ PTEN pathway [138]. Gougelet and his colleagues used a mouse model in which β-catenin signaling was over-activated exclusively in the liver. They found that treatment with an LNA-derived inhibitor of miR-34a remarkably halved progression rates for tumors [69]. As a variety of delivery systems had been applied in HCC gene therapy, liposome-based carrier system was reported to be a potential approach. A new transferrin-targeted delivery system of negatively charged liposomes encapsulating anti-miR-221 was developed and effectively delivered anti-miR-221 to HepG2 cells, which significantly reduced the level of miR-221 [139]. Since previous studies indicated that amiRNA might be a promising therapeutic modality in gene therapy, Huang et al. constructed amiRNAs targeting firefly luciferase with the precursor frameworks of six highly abundant miRNAs in HCC. The results showed that the miR-221 precursor-based amiRNA exhibited a greatest knockdown effect on luciferase activity, indicating that construction of HCC-targeting amiRNAs by the precursor structure of miR-221 could be widely used in HCC treatment [140]. We generated an oncolytic adenoviral vector, which can specifically replicate with high copies in HCC cells, to express an artificially-designed interfering lncRNA (lncRNAi) containing the complementary binding sequences to the seed sequences of the 12 oncogenic miRNAs, including miR-21, miR-221/222, miR-224, miR-17-5p/20a, miR-10b, miR-106b, miR-151-5p, miR-155, miR-181a/181b, miR-184, miR-1 and miR-501-5p. The lncRNAi expressed with high level in HCC cells and competed with target genes of oncogenic miRNAs to bind to and consume those oncogenic miRNAs, thereby achieving the target anti-tumor efficacy on HCC cell line xenograft models and HCC patient-derived xenograft (PDX) models in nude mice [46].

### 4.3. Lung Cancer

Dysregulation of miRNAs contributes to lung carcinogenesis and progression. Fernandez et al. found that the expression of miR-340 was inversely correlated with progression of non-small cell lung cancer (NSCLC). Over-expression of miR-340 suppresses cell growth and induces apoptosis in NSCLC cells [108]. miRNAs are always recognized as potential targets of cancer therapy, and effective delivery strategies still need to be explored. Trang et al. and Ai et al. both reported that delivery of synthetic mimics of suppressor miRNAs in complex with a novel neutral lipid emulsion by blood stream was preferentially targeted to lung tumors and showed remarkable inhibition of tumors in a V-Ki-ras2 Kirsten rat sarcoma viral oncogene homolog (KRAS)-driven mouse models of lung cancer [141,142]. As we know, oncolytic virotherapy is a promising approach for the treatment of advanced NSCLC. Since the expression of miR-145 is lower in NSCLC cells, Li et al. constructed a new oncolytic HSV-1 (AP27i145) carrying four copies of miR-145 target sites in the 3′-UTR of an HSV-1 essential viral gene, infection cell protein 27 (ICP27). AP27i145 replication selectively inhibited the proliferation and neoplastic capacity of NSCLC cells. Moreover, the combination of ionizing radiation and AP27i145 infection was obviously more effective in killing cancer cells than that of monotherapy [54].

### 4.4. Gastric Cancer

Gastric cancer remains one of the most common tumors and affects human health due to its high morbidity and mortality. Studies have revealed that miRNAs are probably associated with tumorigenesis of gastric cancer [143]. Lee et al. identified miR-130a and miR-495 as oncogenic miRNA candidates, both of which are capable of targeting Runt-related transcription factor 3 (RUNX3) and can decrease apoptosis and increase cell proliferation in SNU5 and SNU484 gastric cancer cells. Furthermore, the synthetized antagomirs specific for miR-130a and miR-495 showed strong inhibitory effect on cell growth and angiogenesis [109]. Down-regulation of miR-1 has been reported in gastric cancer. Transfection of miR-1 mimics results in the suppression of cell proliferation and migration. This study provides new insights into target therapy of gastric cancer [144]. In addition, an artificial miRNA targeting liver-intestine cadherin (CDH17) via the lentivirus vector was applied to induce a long-lasting knockdown of CDH17 expression in BGC823 cells, and the CDH17-miRNA-transfected gastric cells showed a significant decrease in cell proliferation, cell motility, and migration in comparison with the control cells [145].

### 4.5. Prostate Cancer

Prostate cancer is one of the leading causes among male cancer-related deaths. Recently, miRNAs have demonstrated as critical post-transcriptional regulators of prostate cancer. Wang et al. reported that transfection of miR-221/222 mimics in prostate cancer cells could increase the activity of cell proliferation and inhibit the pro-apoptotic effect by suppressing caspase-10 [146]. Budd and his colleagues found that inhibition of miR-22 or restoration of miR-125b impaired migratory and invasive potential of prostate cancer cells in vitro [147]. Recent advances in efficient miRNA delivery techniques using prostate cancer-targeted nanoparticles offer critical information for understanding the functional role of miRNAs. Zhang et al. synthesized a polyarginine peptide (R11)-labeled non-toxic disulfidebond polyethylenimine (SS-PEI) nanocarrier for delivery of miR-145 and demonstrated that the systemic administration of R11-SSPEI/FAM-miR-145 complex dramatically inhibited tumor growth and prolonged survival time in a mouse model of intraperitoneally implanting prostate cancer xenografts, without any toxicity [55].

### 4.6. Leukemia

MiRNAs can function either as oncogenes or tumor suppressor genes in leukemia, and open up new opportunities for leukemia therapy. miR-126 was first validated to be a feasible therapeutic target of acute myeloid leukemia (AML). The constructed targeting nanoparticles containing antagomiR-126 can deplete the quiescent cell subpopulation and then reduce the number of leukemia stem cells [148]. Jiang et al. developed a targeted nanoparticle system, FLT3 ligand (FLT3L)-conjugated G7 poly nanosized dendriplex encapsulating miR-150, and demonstrated that the system selectively targets FLT3-overexpressing AML cells and efficiently inhibits cell viability and induces apoptosis both in vitro and in vivo [149]. Similarly, a non-viral system of transferrin (Tf)-conjugated anionic lipopolyplex nanoparticles for miR-29b mimic transfection had many advantages, such as relatively high efficiency of miRNA transfection and low cytotoxicity in AML cells [81]. Mignacca et al. reported that miRNA sponges against miR-19 and miR-155 inhibited the functions of these miRNAs and enhanced the induction of p53 and suppressor of cytokine signaling-1 (SOCS1) in human myeloma cells and mouse leukemia cells, which indicated that the antagonizing miRNA activity could reactivate the activity of cytokine-stimulated tumor suppressor pathways in leukemia cells [119].

## 5. Conclusions

As described above, there have been many new technological advances for utilizing miRNAs as therapeutic tools for cancers. The better understanding of miRNA biogenesis and function undoubtedly affects the research and development of miRNA-based therapies. Until now, several miRNAs have been validated in preclinical tests and left for further clinical investigation. In 2013, the first miRNA replacement therapy with MRX34—a liposome-formulated miR-34 mimic—entered human clinical trials for patients with advanced or metastatic liver cancer by intravenous injection [150,151]. An antagonist of miR-122 was used for hepatitis C treatment and tested in phase II clinical trials [152]. Let-7 mimic was developed to treat a variety of solid carcinomas, such as lung cancer and prostate cancer [91,92]. However, many questions regarding the miRNA-based cancer therapies remain to be overcome, including the suboptimal delivery, low bioavailability, off-target effects or long term safety. Potential employed methods may be the development of some novel miRNA-formulations including nanoparticles, polymers and virus-based approaches. Overall, reprogramming miRNA networks in cancer might constitute numerous reasonable and effective target strategies with a strong potential and chance for success in the war against cancer.

## Figures and Tables

**Table 1:** MicroRNAs (miRNAs) involved in cancers.

MicroRNA	Function	Targets	Experimental Data	Therapeutic Strategy	Reference
miR-145	Tumor suppressor	*ROCK1*, *MMP11*, *Rab27a*, *FSCN-1*, *LASP1*, *MTDH*, *SENP1*, *E2F3*, *MUC13*, *c-Myc*	In vitro experiments in nasopharyngeal, bladder, cervical, lung, liver, breast, gastric, prostate cancer cell lines In vivo experiments in prostate, pancreatic, bladder cancers and multiple myeloma	Mimics Vector-based (viral)	[54,55,56,57,58,59,60,61,62,63,64,65,66,67,68]
miR-34a	Tumor suppressor	*CDK6*, *SIRT1, E2F3*, *c-Met*, *Notch*, *c-Myc*, *Fra-1*, *TPD52*, *c-SRC*, *Bcl-2*, *MYCN*	In vitro experiments in neuroblastoma, glioblastoma and liver, prostate, colon, breast cancer cell lines In vivo experiments in multiple myeloma, glioma and prostate xenografts in mice	Mimics Vector-based (viral)	[69,70,71,72,73,74,75,76,77,78,79,80]
miR-29b	Tumor suppressor	*DNMT3A/3B*, *CDK6*, *MCL-1*, *TCL-1*, *Bcl-2*, *KDM2A*, *MMP2*, *TNFAIP3/A20*, *BCL2L2*	In vitro experiments in glioblastomas, acute myelocytic leukemia (AML), liver, lung, gastric cancer cells In vivo experiments in AML, liver and lung cancers	Mimics Vector-based (viral)	[81,82,83,84,85,86,87,88,89,90]
Let-7a	Tumor suppressor	*K-RAS*, *N-RAS*, *CDK6*, *CDC25A*, *HMGA2*, *MYC*, *RTKN*, *E2F2*	In vitro experiments in lung, gastric, breast and colon cancer cells In vivo experiments in breast and lung cancers	Mimics Vector-based (viral)	[91,92,93,94,95,96,97,98,99,100,101]
miR-340	Tumor suppressor	*ROCK1*, *MYO10*, *MET*, *CDH1*, *NF-x03BA/B1*, *JAK1*, *EZH2*	In vitro experiments in liver, glioma, ovarian, breast, lung cancer cells In vivo experiments in liver cancer	Mimics Vector-based (viral)	[102,103,104,105,106,107,108]
miR495	Tumor suppressor	*MYB*, *Bim-1*, *MTA3*, *JAM-A*, *PRL-3*	In vitro experiments in glioma, AML, lung, breast, gastric, prostate cancer cells In vivo experiments in endometrial, breast, prostate cancers and leukemia	Mimics Vector-based (viral)	[109,110,111,112,113,114,115,116,117]
miR155	Oncogene	*SHIP-1*, *C/EBPβ*, *S0CS1*, *SOCS6*, *FBXW7*, *ZDHHC2*	In vitro experiments in liver cancer and myeloid cells In vivo experiments in pre-B lymphoma/Leukemia and liver cancer	Antisense oligos miR-MASK Sponges	[118,119,120,121,122,123,124,125,126]
miR-21	Oncogene	*PDCD4*, *PTEN*, *TPM1*, *FOXO1*, *Rho-B*, *BTG-2*, *Cdc25A*	In vitro experiments in multiple myeloma, glioblastoma, lung, colon, breast and liver cancer cells In vivo experiments in multiple myeloma	Antisense oligos miR-MASK Sponges or LNA	[127,128,129,130,131,132,133]
